# 23255 Devices Engineered to Collect Exhaled Breath Condensate (EBC) and their Applications

**DOI:** 10.1017/cts.2021.412

**Published:** 2021-03-30

**Authors:** Alexander J. Schmidt, Eva Borras, Anh Nguyen, Nicholas J. Kenyon, Cristina E. Davis

**Affiliations:** 1 Department of Mechanical and Aerospace Engineering, University of California, Davis; 2 Department of Internal Medicine, University of California, Davis

## Abstract

ABSTRACT IMPACT: Human exhaled breath is rich in metabolomic content that represents pulmonary function and gas exchange with blood, which can provide insights into an individual’s state of health. OBJECTIVES/GOALS: Human exhaled breath is rich in metabolomic content that represents pulmonary function and gas exchange with blood. It contains a mixture of compounds that offer insight into an individual’s state of health. Here, we present two novel non-invasive breath sampling devices for use in basic medical practice. METHODS/STUDY POPULATION: The two breath samplers have a disposable mouthpiece, a set of inhale and exhale one-way flap valves to allow condensation of exhaled breath only, and a saliva filter. The housing is constructed out of Teflon®, a chemically inert material to reduce chemical absorbance. The first device condenses exhaled breath into a frozen condensate using dry ice pellets and the other is a miniaturized design that liquifies exhaled breath on a condenser surface with micropatterned features on a cooling plate. Both designs have individual strategic and analytical advantages: frozen exhaled breath condensate (EBC) has high retention of analytes and sample volume; EBC collected in liquid phase offers facilitated sample collection and device portability. RESULTS/ANTICIPATED RESULTS: We investigated if breath aerosol size distribution affects the types or abundances of metabolites. We modified the geometry of the first device to redirect aerosol trajectories based on size. The trapping of larger aerosols increases with filter length, thus altering the aerosol size distribution although no significant changes in the metabolite profiles were found. With the miniaturized device, metabolite abundances were measured in a small cohort of healthy control and mild asthmatic subjects. Differences among subjects were found, as well as main differences between control and asthmatic groups. All analyses of EBC were performed with liquid chromatography - mass spectrometry. Inflammatory suppression found in asthmatic subjects can be explained by prescribed daily use of inhaled corticosteroids. DISCUSSION/SIGNIFICANCE OF FINDINGS: Breath collection devices can be used in intensive care units, outpatient clinics, workplaces, and at home. EBC analysis has been used to monitor asthma and chronic obstructive pulmonary disease. It can be applied to infectious respiratory diseases (e.g. influenza, COVID-19) and for monitoring environmental and occupational chemical exposures.

